# Protective Effects of Melatonin against Severe Burn-Induced Distant Organ Injury: A Systematic Review and Meta-Analysis of Experimental Studies

**DOI:** 10.3390/antiox9121196

**Published:** 2020-11-27

**Authors:** Dewan Md. Sumsuzzman, Jeonghyun Choi, Zeeshan Ahmad Khan, Yonggeun Hong

**Affiliations:** 1 Department of Rehabilitation Science, Graduate School of Inje University, Gimhae 50834, Korea; dewanpavelpharm@gmail.com (D.M.S.); yiopiop0011@nate.com (J.C.); 2 Biohealth Products Research Center (BPRC), Inje University, Gimhae 50834, Korea; acezeeshan@live.com; 3 Ubiquitous Healthcare & Anti-Aging Research Center (u-HARC), Inje University, Gimhae 50834, Korea; 4 Department of Physical Therapy, College of Healthcare Medical Science & Engineering, Gimhae 50834, Korea; 5 Department of Medicine, Division of Hematology/Oncology, Harvard Medical School-Beth Israel Deaconess Medical Center, Boston, MA 02215, USA

**Keywords:** burns, wound healing, melatonin, oxidative stress, inflammation, systematic review, meta-analysis

## Abstract

Extensive burns result in a local wound response and distant-organ injury (DOI) caused by oxidative-stress and inflammation. Melatonin (MT) shows promise in alleviating oxidative-stress and inflammation, but its role in thermal injury is largely unexplored. The present systematic review and meta-analysis were designed to assess the effects of MT on oxidative-stress and inflammatory markers against severe burn-induced DOI. Mean difference (MD)/standard mean difference (SMD) with 95% confidence interval (CI) were estimated using fixed-effect/random-effects models. Eighteen experimental studies met the inclusion criteria. Compared with the control group, MT significantly decreased the levels of malondialdehyde (SMD, −1.03; 95% CI, −1.30, −0.76, *p* < 0.00001) and 4-hydroxynonenal (MD, −1.06; 95% CI, −1.57, −0.56, *p* < 0.0001). Additionally, MT increased the levels of glutathione (SMD, 1.94; 95% CI, 1.27, 2.61, *p* < 0.00001) and superoxide-dismutase (SMD, 0.76; 95% CI, 0.08, 1.45, *p* = 0.03). Finally, MT significantly decreased the levels of tumor necrosis factor-α (SMD, −1.34; 95% CI, −1.92 to −0.77; *p* < 0.00001) and C-reactive protein (MD, −12.67; 95% CI, −16.72 to −8.62; *p* < 0.00001). Meta-analysis indicates that severe burn followed by immediate MT (10 mg/kg) intervention shows significant beneficial effects after 24-h against DOI by regulating oxidative-stress and the inflammatory response.

## 1. Introduction

Burns represent complex traumatic skin injuries caused mainly by heat, radiation, electricity, abrasion, or exposure to chemicals [1]. In 2004, approximately 11 million people worldwide suffered burns [2], and the estimated average healthcare cost in developed countries was $88,218/patient [3]. According to the American Burn Association, about 72% of burns are thermal (41% from flames and 31% from scalds) [1], and the fire-related mortality rate is higher in developing countries than in developed nations [4,5]. Although recent advances in the management of severe burn care have improved significantly, multiple organ failure (MOF) is still considered a leading cause of mortality and morbidity following burn injuries, in particular when at least 30% of total body surface area (TBSA) burned [6].

Burn injuries is a critical care crisis, which not only damages the skin locally but also exerts detrimental effects on distant organs and can lead to MOF [7,8,9,10]. The development of complications and poor outcomes after burn injuries is often related to inflammation, blood coagulation disorders, hemorrhagic changes, and oxidative stress [8,9,10,11,12,13,14,15,16]. Additionally, the burn-induced inflammatory response creates a high demand for fluid resuscitation owing to excessive protein and fluid leakage into the interstitial spaces [8]. Accumulating evidence demonstrates that the inflammatory response and oxidative stress contribute to the progression of distant organ injury (DOI) following severe burns [8,11,12,13]. However, the pathophysiological mechanism underlying burn-induced DOI remains elusive. Oxygen radicals induce a local wound response and promote organ impairment, including of the liver [10,17], gastric mucosa [18], intestine [19,20], heart [21,22], and lung [23,24]. Burn injuries promote lipid peroxidation, an autocatalytic mechanism leading to oxidative damage to cellular membranes, the release of toxic, reactive metabolites, and cell death [25,26]. Likewise, Youn et al. [27] reported that burn injuries increase lipid peroxidation in plasma, liver, and lung. Because the oxidizing agent may originate from neutrophils sequestered in systemic organs as a systemic inflammatory reaction to a local burn insult [28], agents that suppress neutrophil activation and adherence might protect against thermal injury [29]. Moreover, antioxidant molecules reduce the excessive demand for fluid resuscitation [30]. Antioxidants are administered during the post-burn period to restore the oxidant-antioxidant balance and attenuate the inflammatory response, blood coagulation, and tissue injury [15,31,32]. Despite this knowledge, it is surprising that still, the clinical application of anti-oxidant therapy as an adjunct to burn care is limited [33]. In 2016, a systematic review by Adjepong et al. [34] reported that so far, only eleven studies devoted to the efficacy of micronutrients anti-oxidant therapy in burn patients had been published. Although this study reported the beneficial effects of antioxidant supplements in burn patients, the dose, timing, and the duration of the antioxidant use remain unclear. In addition, to date, only a limited number of antioxidants have been identified for the management of burn care [34,35]. The area of anti-oxidant therapy to prevent consequences of severe burn-induced DOI represents a virtually unexplored area that holds promise for effective adjunctive therapy. Hence, evidence synthesis is urgently required to study the role of anti-oxidants in burn injury, including potent antioxidants with anti-inflammatory, and immunomodulatory biomolecule like melatonin [36,37,38].

Melatonin (MT), a neurohormone produced mainly in the pineal gland, is a potent free radical scavenger and antioxidant. It scavenges reactive oxygen species (ROS) and reactive nitrogen species (RNS), stimulates the activity of antioxidant enzymes, inhibits proinflammatory cytokines, and activates adhesion molecules [39,40,41]. MT can preserve the level of glutathione (GSH) within cells and in mitochondria, suppressing oxidative damage [42,43]. MT not only attenuates oxidative damage in experimental burns [44,45,46,47,48] but also in burn patients [49]. While previous research indicates that MT could be beneficial in instances of DOI after severe skin burn, a comprehensive review on this topic has yet to be performed. Besides, MT shows promise in alleviating oxidative stress and inflammation following thermal injury in animals, the clinical use of MT is mostly limited to sleep-related outcomes, obesity [50,51], and dental diseases [52,53]. Therefore, we conducted a systematic review and meta-analysis to investigate the experimental data that support the clinical applicability of MT in the treatment of burn-induced DOI, with particular emphasis on oxidant-antioxidant balance and reduction of inflammation.

## 2. Materials and Methods

### 2.1. Search Strategy

To identify potentially relevant studies, a two-step systematic literature search was conducted using the Preferred Reporting Items for Systematic Reviews and Meta-analyses (PRISMA) guidelines [54]. Firstly, for comprehensive literature searching, the PubMed, Embase, and CINAHL electronic databases were searched for studies that assessed the effects of exogenous MT on oxidative stress and inflammatory markers in burn wound animal models up to August 2020. To identify additional relevant articles, the reference lists of identified studies were manually scanned. We used filters to retrieve only animal studies from PubMed and Embase. No limits (e.g., on language or publication date) were used.

The full search strategies for the Pubmed was: (“wound healing”[MeSH Terms] OR “wound healing”[Text Word] OR “wound healings”[Text Word] OR “wound repair”[Text Word] OR “wound*”[Text Word] OR “scar”[Text Word] OR “scar*”[Text Word] OR “thermal burn”[Text Word] OR “thermal injury”[Text Word] OR “burns”[MeSH Terms] OR “burn*”[Text Word] OR “cicatrix”[MeSH Terms] OR “cicatri*”[Text Word] OR “granulation tissue”[MeSH Terms] OR “granulation tissue”[Text Word] OR “reepithelialization”[Text Word]) AND (“melatonin”[MeSH Terms] OR “receptors, melatonin”[MeSH Terms] OR “tasimelteon”[Supplementary Concept] OR “ramelteon”[Supplementary Concept] OR “S-20098”[Supplementary Concept] OR “melatonin*”[Text Word] OR “tasimelteon”[Text Word] OR “ramelteon”[Text Word] OR “S-20098”[Text Word] OR “S20098”[Text Word] OR “circadin”[Text Word] OR “rozerem”[Text Word] OR “hetlioz”[Text Word] OR “valdoxan”[Text Word] OR “thymanax”[Text Word] OR “Melovine”[Text Word] OR “5-methoxy-n-acetyltryptamine”[Text Word] OR “n-acetyl-5-methoxytryptamine”[Text Word] OR “MT”[Title/Abstract] OR “MLT”[Title/Abstract]).

In Embase, the full search string was used: (‘wound healing’/exp OR ‘wound healing’:ab,ti,kw OR (granulation:ab,ti,kw AND wound:ab,ti,kw) OR (healing:ab,ti,kw AND wound:ab,ti,kw) OR (repair:ab,ti,kw AND wound:ab,ti,kw) OR (wound:ab,ti,kw AND regeneration:ab,ti,kw) OR (wound:ab,ti,kw AND repair:ab,ti,kw) OR (lenticular:ab,ti,kw AND wound:ab,ti,kw) OR ‘vulnus’/exp OR (wound:ab,ti,kw AND age:ab,ti,kw) OR ‘wound’/exp OR wound:ab,ti,kw OR ‘wounding’/exp OR ‘scar’/exp OR scar:ab,ti,kw OR ‘cicatrix’/exp OR cicatrix:ab,ti,kw OR (radiation:ab,ti,kw AND scar:ab,ti,kw) OR ‘burn’/exp OR burn:ab,ti,kw OR (burn:ab,ti,kw AND complication:ab,ti,kw) OR (burn:ab,ti,kw AND injury:ab,ti,kw) OR (burn:ab,ti,kw AND trauma:ab,ti,kw) OR (burn:ab,ti,kw AND wound:ab,ti,kw) OR ‘burning’/exp OR ‘burns’/exp OR (deep:ab,ti,kw AND burn:ab,ti,kw) OR (skin:ab,ti,kw AND burn:ab,ti,kw) OR (thermal:ab,ti,kw AND burn:ab,ti,kw) OR ‘granulation tissue’/exp OR (tissue:ab,ti,kw AND granulation:ab,ti,kw) OR ‘reepithelialization’/exp OR reepithelialization:ab,ti,kw) AND (‘melatonin’/exp OR ‘melatonin’:ab,ti,kw OR ‘melatonin receptor’:ab,ti,kw OR ‘tasimelteon’:ab,ti,kw OR ‘ramelteon’:ab,ti,kw OR (s:ab,ti,kw AND 20098:ab,ti,kw) OR melatonin*:ab,ti,kw OR ‘s 20098′:ab,ti,kw OR s20098:ab,ti,kw OR ‘tik 301′:ab,ti,kw OR tik301:ab,ti,kw OR circadin:ab,ti,kw OR ‘ly 15635′:ab,ti,kw OR rozerem:ab,ti,kw OR hetlioz:ab,ti,kw OR valdoxan:ab,ti,kw OR thymanax:ab,ti,kw OR melitor:ab,ti,kw OR melovine:ab,ti,kw OR ‘5 methoxy n acetyltryptamine’:ab,ti,kw OR ‘n acetyl 5 methoxytryptamine’:ab,ti,kw OR ‘vec 162′:ab,ti,kw OR vec162:ab,ti,kw OR ‘tak 375′:ab,ti,kw OR tak375:ab,ti,kw OR mt:ab,ti,kw OR mlt:ab,ti,kw).

### 2.2. Inclusion and Exclusion Criteria

The following inclusion criteria were applied to study selection (Supplementary Table S1): (1) animal models of burn wounds; (2) any type of MT treatment compared with a placebo control; (3) a control intervention of saline, ethyl alcohol, or other vehicle; and (4) anti-oxidative and anti-inflammatory effects of MT in an animal model with severe burn-induced DOI. The following exclusion criteria were applied to the study selection (Supplementary Table S2): (1) clinical case reports, ex vivo, and studies solely in vitro, (2) non-original studies (e.g., editorials or literature reviews), (3) studies not freely available, and (4) conference abstracts.

### 2.3. Study Selection

After the removal of duplicates, all unique trials were imported into the Rayyan-a web application to allocate the references randomly [55]. Next, two of the authors independently screened the titles and abstracts to select relevant studies from the randomly allocated references. It should be noted that we did not screen for the presence or absence of specific outcome measures during this phase because, often, not all outcome measures were described in the abstract. Finally, the full-texts of the selected articles were evaluated to identify those that fulfilled the inclusion criteria. Any disagreement concerning study selection was settled by consultation with the third author.

### 2.4. Data Extraction

Two authors independently extracted from each of the included studies information on the authors, publication year, species, weight, sample size, animal model, intervention (dose and administration time), outcome measures, and mean and SD of each oxidative and/or inflammatory marker (for both MT-treated and controls). In studies with multiple interventions, only data from the control and MT experimental groups were considered in the analysis. If the published outcome data were incomplete, we attempted to contact the authors to obtain the original data. A reminder was sent by email to those who had not responded within 2 weeks. If efforts to achieve the original data failed, the article was eliminated from the meta-analysis. If the data were presented graphically, GetData Graph Digitizer (http://getdata-graph-digitizer.com/) was employed to extract numerical data from graphs or figures.

### 2.5. Assessment of Methodological Quality

The risk of bias (RoB) in the included articles was evaluated by two independent reviewers using the SYRCLE RoB tool [56]. Based on the Cochrane RoB tool [57], the RoB tool was developed to evaluate the aspects of bias specific to animal intervention studies. The tool contains 10 items related to six types of bias (selection, performance, detection, attrition, reporting, and other). Scores of ‘yes’, ‘no’, and ‘unsure’ indicate a low, high, and unclear RoB, respectively.

### 2.6. Data Analysis

The experimental and control group data from the included studies were extracted and input into Review Manager Software (ver. 5.3, The Nordic Cochrane Centre, Copenhagen, Denmark). The meta-analysis was executed when a minimum of two studies were analogous in terms of the population, intervention, comparison, outcomes, and design, and provided relevant data. In the effect-size analysis, the mean difference (MD) was used when the outcome measure of all studies employed the same scale, whereas the standardized mean difference (SMD) was used when the studies assessed the same outcome but measured it in different ways. For both strategies, 95% confidence intervals (CIs) were calculated. The I^2^ test was utilized to assess heterogeneity among the studies. The fixed-effects model was used for the meta-analysis when I^2^ was ≤50% and the random-effects model when I^2^ was >50% (indicative of substantial heterogeneity) [58]. Subgroup analyses were performed only if the subgroups contained a minimum of two independent comparisons. To determine the influence of each study on the overall effect size, a sensitivity analysis was conducted using the leave-one-out approach [59]. To generate the leave-one-out forest plot, we used OpenMeta [Analyst] software (http://www.cebm.brown.edu/openmeta/).

### 2.7. Publication Bias

Based on the Cochrane recommendations, we analyzed funnel plot asymmetry when one outcome variable was associated with at least 10 studies in the meta-analysis, since with <10 studies the power of the tests is too low [60]. Potential publication bias was assessed by visual inspection of funnel plot asymmetry and Egger’s test of asymmetry [61]. Whenever asymmetry was detected in the funnel plot, the trim and fill method was used to calculate the effect size by estimating the number of missing studies [62]. All statistical analyses related to publication bias were carried out in JASP, an open-source statistical program developed by the University of Amsterdam (https://jasp-stats.org/).

## 3. Results

### 3.1. Study Selection

**Figure 1:**
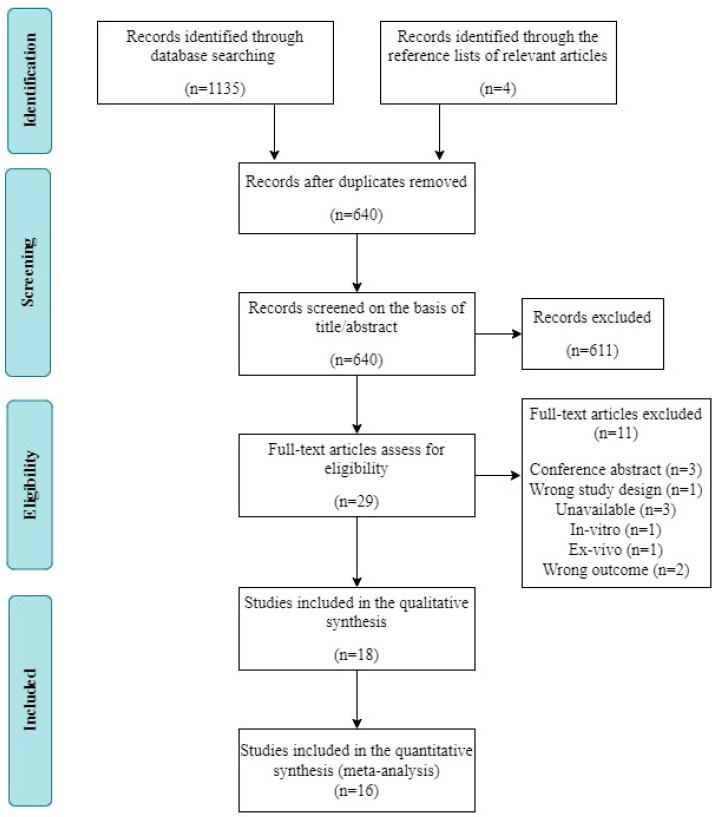
Flow diagram of the systematic review and literature search results.

A total of 1135 studies were identified via electronic database searches. After filtering out duplicate studies, the titles and abstracts of 640 potentially relevant articles were screened. Of these articles, 611 were excluded. The remaining 29 studies were subjected to full-text screening. Among them, 11 studies were excluded for the following reasons: conference abstract (n = 3), inappropriate study design (n = 1), unavailable data (n = 3), in vitro design (n = 1), ex vivo design (n = 1), and inappropriate outcome measure (n = 2). Ultimately, 18 studies fulfilled the inclusion criteria and were included in the systematic review; data could not be extracted from two of those studies, leaving 16 records available for our meta-analysis (Figure 1).

### 3.2. Study Characteristics

The main characteristics of the 18 included studies are listed in Table 1. Among these studies, the sample size per group ranged from 3 to 19. Eight studies focused on hepatic injury [63,64,65,66,67,68,69,70], four on gastric mucosal damage [45,71,72,73], two on kidney injury [74,75], two on plasma [76,77], one on intestine injury [78], and one on lung injury [79]. All of the studies used hot water (90–100 °C) to induce burn wounds in rats. Most of the studies involved burns to 30% of the total body surface area (TBSA), although one study [69] involved burns to 20% of the TBSA, and another to 40% of the TBSA [75]. The exposure time ranged from 10–15 s.

### 3.3. Risk of Bias and Quality of Reporting

**Figure 2:**
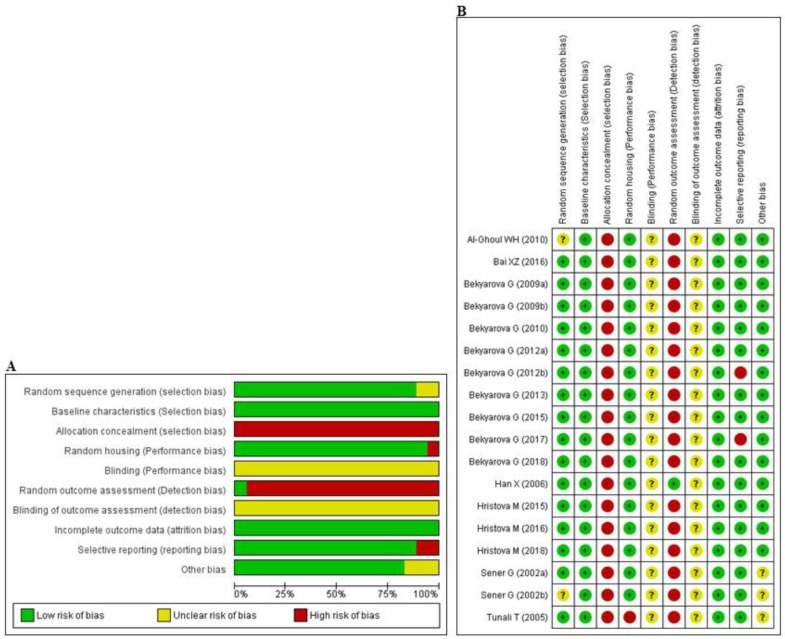
Risk of bias. (**A**) Overall risk of bias for each item in the SYRCLE tool for all included studies. Each risk of bias item is presented as a percentage based on all included studies. (**B**) Individual risk of bias for each of the included animal studies. Each item in the SYRCLE tool was scored as ‘yes’, ‘no’, or ‘unclear’.

The abridged risk of bias (RoB) assessment is presented in Figure 2A, and the individual RoB scores of each study are shown in Figure 2B. Experimental animals in most of the studies had randomly allocated, and maintained under unified room temperature, humidity, and food supply. However, allocation concealment and random outcome assessment were not described, resulting in a high risk of selection and detection bias, respectively, in all studies. In addition, the issue of selective reporting was not discussed in two studies [64,68], suggesting that they may have had a high level of reporting bias. All of the studies reported that the animals were of similar weight and underwent the same experimental design at baseline. Inadequate reporting of the measures used to decrease bias was captured by our RoB assessment; in numerous cases, the measures used were unclear.

### 3.4. Data Analysis

#### 3.4.1. Effect of MT on Oxidative Stress Markers

**Figure 3:**
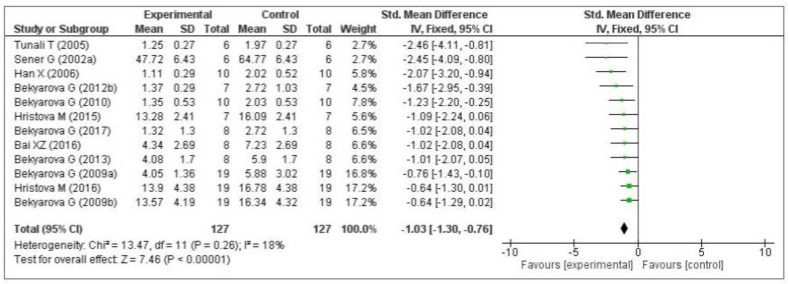
Forest plot showing the impact of MT on MDA levels. MDA, malondialdehyde; CI, confidence interval; IV, independent variable.

Thirteen of the included studies measured the malondialdehyde (MDA) level. Of these 13 studies, 1 [74] did not provide the number of samples per group and was therefore eliminated from the meta-analysis. Of the remaining 12 studies, five measured the MDA level in the liver [64,66,68,69,70], three in the gastric mucosa [71,72,73], one in the kidneys [75], two in the plasma [76,77], and one in the lungs [79]. The data of the 12 eligible studies are shown in Figure 3. Compared with the control group, MT significantly decreased the level of malondialdehyde (standardized mean difference [SMD], −1.03; 95% confidence interval [CI], −1.30, −0.76, *p* < 0.00001). This effect size was robust according to the sensitivity analysis, i.e., the omission of individual studies did not abolish the significance (Supplementary Figure S1).

**Figure 4:**
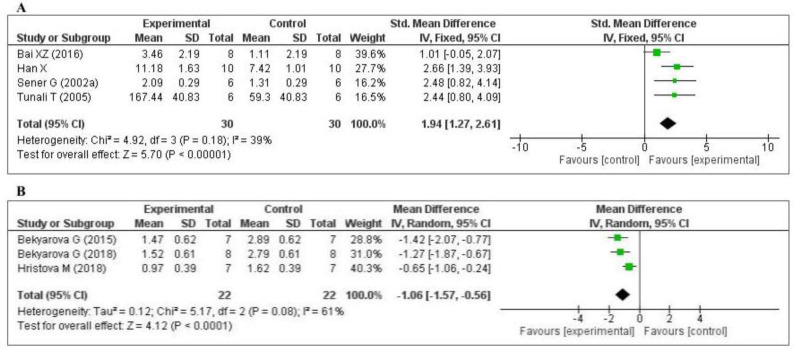
Forest plot showing the impact of MT on GSH and 4-HNE levels ((**A**) GSH; (**B**) 4-HNE). GSH, glutathione; 4-HNE, 4-hydroxynonena; CI, confidence interval; IV, independent variable.

Five studies [70,74,75,77,79] measured the glutathione (GSH) level: one in the liver, two in the kidney, one in the lung, and one in the plasma. Of the five studies, one [74] did not provide the number of samples per group and was eliminated from the meta-analysis. The data of the four studies are shown in Figure 4A. MT increased the level of glutathione (SMD, 1.94; 95% CI, 1.27, 2.61, *p* < 0.00001). Three studies [45,63,65] measured the tissue 4-hydroxynonenal (4-HNE) level: two in the liver and one in the gastric mucosa. The data of the three studies are shown in Figure 4B. MT significantly decreased the level of 4-HNE (mean difference [MD], −1.06; 95% CI, −1.57, −0.56, *p* < 0.0001). The effect sizes were robust according to the sensitivity analysis, i.e., the omission of individual studies did not abolish the significance (Supplementary Figures S2 and S3). Thus, when compared with the control group, MT-treatment was significantly increased GSH level and decreased 4-HNE level.

**Figure 5:**
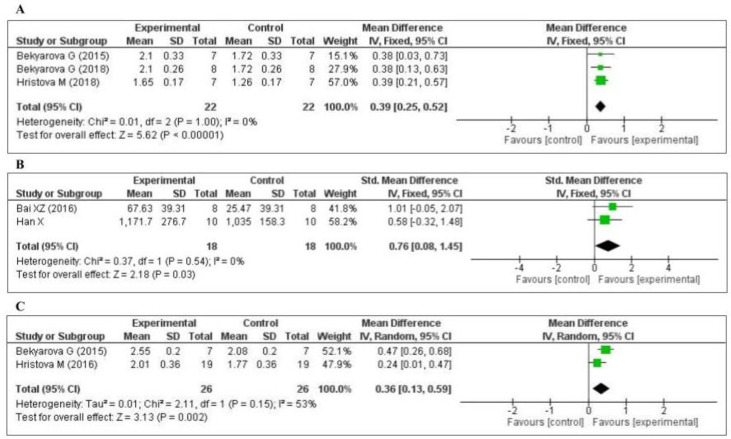
Forest plot showing the impact of MT on Nrf2, SOD, and HO-1 levels ((**A**) Nrf2, (**B**) SOD, (**C**) HO-1). Nrf2, nuclear factor erythroid 2-related factor 2; SOD, superoxide dismutase; HO-1, heme oxygenase-1, CI, confidence interval; IV, independent variable.

Three studies [45,63,65] measured the nuclear factor erythroid 2-related factor 2 (Nrf2) level: two in the liver and one in the gastric mucosa. The data of the three studies are shown in Figure 5A. MT increased the level of Nrf2 (MD, 0.39; 95% CI, 0.25, 0.52, *p* < 0.00001). Two studies [75,79] measured the tissue superoxide dismutase (SOD) level: one in the kidney and one in the lung. The data of the two studies are shown in Figure 5B. MT significantly decreased the level of SOD (SMD, 0.76; 95% CI, 0.08, 1.45, *p* = 0.03). Two studies [65,71] measured the tissue heme oxygenase-1 (HO-1) level: one in the liver and one in the gastric mucosa. The data of the two studies are shown in Figure 5C. MT increased the level of HO-1 (MD, 0.36; 95% CI, 0.13, 0.59, *p* = 0.002). The effect sizes were robust according to the sensitivity analysis, i.e., the omission of individual studies did not abolish the significance (Supplementary Figures S4–S6).

#### 3.4.2. Effect of MT on Inflammatory Markers

**Figure 6:**
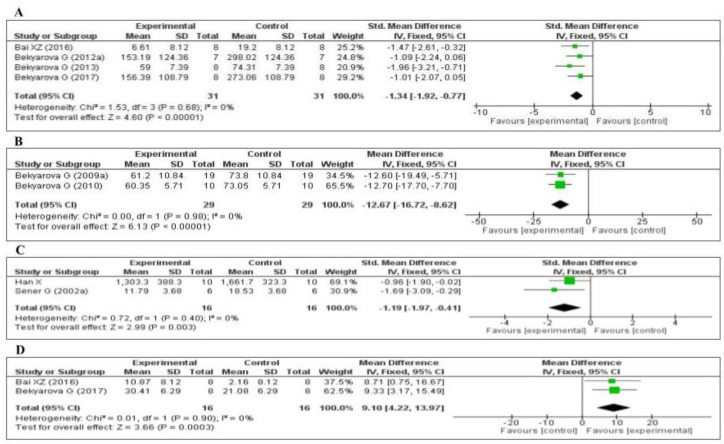
Forest plot showing the impact of MT on various inflammatory markers ((**A**) TNF-α, (**B**) CRP, (**C**) MPO, (**D**) IL-10). TNF-α, tumor necrosis factor-α; CRP, C-reactive protein; MPO, myeloperoxidase, IL-10, Interleukin-10, CI, confidence interval; IV, independent variable.

Four of the included studies measured the tumor necrosis factor-α (TNF-α) level: three [64,66,67] in the liver and one [75] in the kidney. The data of the four studies are shown in Figure 6A. MT significantly decreased the level of TNF-α (SMD, −1.34; 95% CI, −1.92, −0.77, *p <* 0.00001). Two of the studies measured the plasma C-reactive protein (CRP) level [69,76]. The data of the two studies are shown in Figure 6B. MT significantly decreased the level of CRP (MD, −12.67; 95% CI, −16.72, −8.62, *p* < 0.00001). Two of the studies measured the myeloperoxidase (MPO) level [70,79]. The data of the two studies are shown in Figure 6C. MT significantly decreased the level of MPO (SMD, −1.19; 95% CI, −1.97, −0.41, *p* = 0.003). Finally, two of the studies measured the tissue interleukin-10 (IL-10) level [64,75]: one in the liver and one in the kidney. The data of the two studies are shown in Figure 6D. MT significantly decreased the tissue level of IL-10 (MD, 9.10; 95% CI, 4.22, 13.97, *p* = 0.0003). The effect sizes were robust according to the sensitivity analysis, i.e., the omission of individual studies did not abolish the significant effect (Supplementary Figures S7–S10).

#### 3.4.3. Effect of MT on Other Inflammatory Markers

Nuclear factor κB (NF-κB), a transcriptional factor, is activated immediately after severe burn injury and is thought to regulate the expression of several inflammatory mediators, including TNF-α [80]. It has previously been observed that an increase the expression of TNF-α further stimulates for the production of several secondary cytokines, which is considered a crucial pathophysiological mechanism of liver injury following severe burns [81,82]. Interestingly, the liver is one of the most prone organs to inflammatory damage in thermal injury [83]. It is hypothesized that MT decrease severe burn-induced liver damage by inhibiting NF-κB activation. In this systematic review, we found one study that assessed the effects of MT on NF-κB expression in the liver of third-degree burn rats [67]. The findings of this study disclosed that the TNF-α level increased with hepatic NF-κB expression in the burned group. However, MT treatment significantly decreased not only in the hepatic TNF-α level but also reduced NF-κB expression in the liver. These results indicating elevated NF-κB expression triggers hepatic TNF-α production, which further stimulates liver NF-κB activation.

Following heat insult, the inducible nitric oxide synthase (iNOS) acts as a downstream mediator of inflammation in various organs, including the stomach [45,84]. Previous research has established that a significant reduction of iNOS expression is associated with decreased systemic inflammation and oxidative stress in burn injury [19]. Despite the comprehensive search strategy, we identified only one study, which examined the effects of MT on iNOS expression in gastric mucosa [71]. In this study, iNOS expression was markedly elevated in gastric mucosa after severe thermal injury. In contrast, MT restricted the increased iNOS (*p* < 0.05) levels in gastric mucosa [71].

Most recently, Guo et al. [85] reported that compared with iNOS deficient burned mice, interleukin-1β (IL-1β) mRNA expression gradually increased with elevated iNOS levels in wild-type burned mice. This finding is supported by Yuan and peers study who disclosed that pro-inflammatory cytokines such as IL-1β began to increase following thermal injury, which responsible for various organ damage, including kidney [86]. According to our inclusion criteria, we included one study that evaluated the effects of MT on IL-1β levels in post-burn induced acute kindly injury [75]. This study demonstrated that severe burns could increase IL-1β levels in renal tissues, which could be decreased by MT treatment.

Disruption of the intestinal barrier plays a pivotal role in the pathophysiology of severe burn injury [87]. Although molecular mechanisms of severe burn-induced intestinal barrier disruption are not well-understood yet, extensive research is ongoing to elucidate its pathophysiology. However, recent evidence suggests that intestinal inflammation may contribute to intestinal barrier disruption [88,89]. Nitrotyrosine, a biomarker of inflammation [90,91,92], can increase with iNOS expression in full-thickness third-degree burn injury (40% TBSA burn) [93]. On the other hand, the nitrotyrosine contents are markedly reduced by the iNOS inhibitor [93]. Interestingly, we found one study that shows nitrotyrosine levels was peak in ileum tissue of post-burn rats, whereas this marker was apparent significantly decreased in MT treated rats [78].

#### 3.4.4. Assessment of Publication Bias

**Figure 7:**
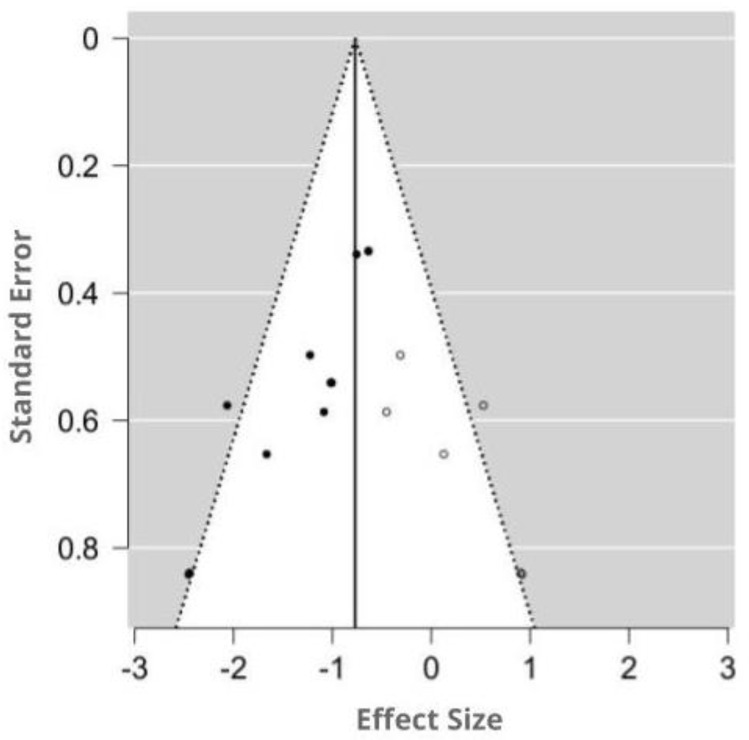
Funnel plot showing the publication bias in studies reporting the impact of MT on MDA levels. Open circles represent published studies; closed circles represent unpublished studies.

The funnel plot was asymmetry by visual inspection, and the significant publication bias was detected by Egger’s test (*p* = 0.001). Using the trim-and-fill method, six potentially missing studies were imputed for the analysis of MDA. The imputed effect size of MDA was SMD −0.77 (95% CI −1.01, −0.53). A funnel plot of the impact of MT on MDA activity is shown in Figure 7.

## 4. Discussion

### 4.1. Main Findings

The results of this systematic review and meta-analysis demonstrated that exogenous MT effectively regulated oxidative stress and inflammatory markers in different distant organs including, liver, gastric mucosa, lung, and kidney in animal models. Sixteen studies were eligible for meta-analysis, which showed that MT significantly reduced the MDA and 4-HNE levels and increased the GSH, Nrf2, SOD, and HO-1 levels. Therefore, MT decreased the levels of lipid peroxidation products and increased those of antioxidant enzymes and of the transcription factors that regulate the synthesis of antioxidant proteins. Furthermore, MT significantly decreased the TNF-α, CRP, and MPO levels and increased the IL-10 level. These findings highlight the therapeutic potential of MT for burn-induced injury to distant organs.

### 4.2. Strengths and Limitations

To our knowledge, this is the first systematic review of the effects of MT on burn wound-induced damage to distant organs. We applied a comprehensive search strategy to multiple databases, had access to the full texts of all identified studies, used the SYRCLE RoB tool to assess the methodological quality of the studies, and extracted data pertaining to the levels of a wide range of oxidative stress and inflammatory markers. Furthermore, we performed a sensitivity analysis to validate our findings. Whenever asymmetry was detected in the funnel plot, the trim and fill method were used to adjust the effect size according to the number of missing studies.

This study also had several limitations. First, we assessed the effects of MT on antioxidant and anti-inflammatory activities 24 h after a burn injury; the early effects were not assessed. Second, during full-text screening, we had to exclude many studies that were not freely available. Third, quality assessment using the SYRCLE RoB tool indicated that all of the included studies had significant methodological limitations and a high risk of selection and detection bias. Finally, although we contacted the study authors to obtain missing data, the data were not provided.

### 4.3. Clinical Importance

The limitations of the data notwithstanding, the results indicate that MT prevents injury to distant organs by ameliorating oxidative stress and inflammation. This finding is consistent with previous reports [70,73,75]. Multiple organ dysfunction syndromes (MODS) can develop in patients with extensive burns [94,95]. Huang [96] showed that the levels of all inflammatory mediators markedly increased in animals and patients who sustained organ damage or MODS. Our finding is also in agreement with the report by Huang [96] that severe burns increased the levels of inflammatory mediators in distant organs and that MT significantly decreased the TNF-α, CRP, and MPO levels and increased those of anti-inflammatory cytokines such as IL-10. Recently, Lv et al. [97] demonstrated that hepatic NF-κB and TNF-α expression were upregulated after 30% of full-thickness burns, suggesting elevated NF-κB expression associated with activation of inflammatory response. In line with these findings, our results also indicate that MT treatment reduced the hepatic TNF-α level by downregulation of NF-κB expression following thermal injury. We also narratively presented that the iNOS expression was upregulated following the post-burn, on the other hand, MT intervention significantly reduced the iNOS levels. This finding is contrary to previous studies which have reported that there is no significant difference between control and burn injury groups [98]. A possible explanation for this discrepancy might be that the iNOS expression may be time-dependent in burn injury. More clearly, iNOS related inflammation obviously appears in the initial stage of thermal injury [99], not later stages. Furthermore, oxidative stress is one of the main causes of pathophysiological alterations during burn injury. Exogenous antioxidant therapy reportedly prevents oxidative injury experimentally and clinically [100,101]. Administration of an antioxidant to rats markedly inhibited the increase in the levels of lipid peroxides in the plasma, lung, and kidney and decreased the plasma protein level, particularly at the early stage of burn injury [100]. Indeed, we found that MT not only significantly reduced the levels of lipid peroxidation products (including MDA and 4-HNE) but also increased the levels of antioxidant enzymes such as GSH, SOD and HO-1. Furthermore, the activation of Nrf2 protects cells from oxidative stress and inhibits the redox-mediated inflammatory response [102]. For example, Braun et al. reported that Nrf2 modulates gene expression and the inflammatory response during healing of skin wounds [103]. In recent several studies further reported that Nrf2 and antioxidant enzymes (SOD, HO-1) were upregulated by MT treatment, suggesting MT promotes the translocation of Nrf2 from the cytoplasm to the nucleus and increased Nrf2 mediated antioxidant enzymatic activities [104,105]. In the same vein, our meta-analysis also demonstrated that MT significantly increased the Nrf2 level in severely burn-induced experimental rats.

### 4.4. Implications for Future Research

In reviewing the existing literature, it is assumed that oxidative stress not only directly damages the tissue following the thermal injury of the skin but also it activates an inflammatory response that combinedly contributes to MOF. These findings support the idea that antioxidant therapy might be useful in post-burn injury to protect against DOI, especially when TBSA greater than 20%. To date, several antioxidant molecules have been proposed for restoring the antioxidant defenses and reduce mortality after thermal injury. Even some of them have already shown beneficial effects in both animal [106] as well as human studies [107]. However, the clinical application of antioxidants in burn injury is limited. For example, although MT, widely known as an antioxidant, shows salutary effects in experimental burn injury, the clinical use of MT is only restricted to sleep-related outcomes (NCT01598259). Last but not least, along with antioxidative effects, MT also reduces the inflammatory response in 30% TBSA burned rats. Although these combined effects of MT would make it comparatively highly attractive as an add-on therapy for the management of progressive organ failure after burn injury, the pharmacokinetics of MT has not been reported in burns. Maybe due to the lacking of pharmacokinetics data, the applicability of MT in burn injury is only restricted to sleep-related outcomes. Further studies, which take these variables into account, will need to be undertaken.

Another critical issue that is still unclear is whether a single antioxidant intervention is enough for optimal outcomes in burn injury or a combination of antioxidants is necessary. Preliminary, ascorbic acid (vitamin-C) has shown the beneficial effects regarding the improvement of wound healing and reduction of ventilation requirements in severe burns patients [108]. However, to achieve these goals, a high dose of ascorbic acid (66 mg/kg and maintenance dose 33 mg/kg/h) administration is required; unless otherwise, half of the dose is practically ineffective [109,110]. In a randomized controlled trial, high-dose of ascorbic acid causes oxalate crystallization, stone formation, and nephropathy in susceptible patients [111]. In addition, gender is considered one of the risk factors for ascorbic acid associated renal insufficiency. For example, >1000 mg/day is not related to renal stone formation in females, surprisingly, 700 mg/day of the ascorbic acid dose is enough to form stones in males [112]. To overcome this problem, a combination of ascorbic acid with other antioxidants like MT might be an attractive alternative strategy for the management of burn care. Because of the unique strength of MT to improve sleep parameters against severe burns, together with its low toxicity and its capability to reduce inflammation and oxidative stress, are all significant to its efficiency for protecting severe burn mediated distant organ damage. It has already proven that high-dose and prolonged MT treatment did not associate with any adverse effects in different human studies [113,114]. Recently, numerous studies evidence that MT and ascorbic acid combinedly express synergistic effects in several oxidative stress-induced disease conditions [115,116,117]. Interestingly, one recent study reported that ascorbic acid is less effective than MT to improve stress-induced gastric mucosal damage [118]. Thereby, we may assume that the combination of MT and ascorbic acid or other antioxidants, together not only enhances the burn injury recovery process, but also MT may cut-off the higher dose of ascorbic acid. There is abundant room for further progress in determining the efficacy of this combination in thermal injury. Further work is also required when MT is given in combination with other drugs for the treatment of burn injury. Taken together, MT may reduce the adverse effects of antioxidants and/or other drugs that currently used to treat burn patients, consequently will increase their efficacy.

Last but not least, in recent years, population-based studies reported that both severe and mild thermal injury leading to long-term health effects, including acute kidney injury [119], respiratory infection [120], circulatory system diseases [121], hepatic dysfunction [122]. Although the clinical management of burns has improved significantly, resulting in gradually increase in survival rates, there is growing evidence of longtime impacts of burn injury. Recent precedents suggest burn injury is linked to continuous changes to immune function [123], which contribute to multiple organ damage. In this context, MT would be an ideal candidate to stimulate immune function for long-term burn care because it has inherent immunomodulatory effects [124,125,126]. In our review, MT has been highlighted as a potential therapeutic candidate for short-term burns care, however; the data of long-term effects of this pineal hormone for the management of burn care are absent. Future studies are required to assess the impact of MT on the long-term burden of burn injury.

## 5. Conclusions

In conclusion, we found that MT treatment significantly reduced lipid peroxidation products (MDA and 4-HNE) and inflammatory molecules (TNF-α, CRP, and MPO) in burn-induced DOI. Additionally, our findings demonstrate that MT may exert a protective effect by increasing the levels of GSH, SOD, and HO-1 via Nrf2 activation. Moreover, MT treatment notably increased the level of anti-inflammatory molecules (e.g., IL-10) in damaged tissues. Combining the attributes of an antioxidant and an anti-inflammatory agent, MT could ameliorate DOI following thermal skin injury. However, all in vivo studies to date have major methodological limitations, including a small sample size, and detection and selection biases. Therefore, future studies should aim to enhance quality through precise experimental design by improving the sample size and reducing the bias in animal trials.

## Figures and Tables

**Table 1:** Characteristics of the included studies.

Author (year)	Animal Characteristics			Study Characteristics			Intervention Characteristics		Outcome Measures
	Species (Gender)	Weight (gm)	Model (Exposure Time)	Exp Group (n)	Con Group (n)	Study Area	MT Use Time	MT in Dose	
Bekyarova G (2018) [63]	Rat (Male)	220–250	30% of TBSA burns by 90 °C hot water (10 s)	B + MT (8)	B + MT (8)	Liver	Immediately after 30% of TBSA burns	10 mg/kg	4-HNE, Nrf2
Bekyarova G (2017) [64]	Rat (Male)	220–250	30% of TBSA burns by 98 °C hot water (10 s)	B + MT (8)	B + V (8)	Liver	Immediately after 30% of TBSA burns	10 mg/kg	TNF-α, IL-10, MDA
Bekyarova G (2015) [72]	Rat (Male)	220–250	30% of TBSA burns by 90 °C hot water (10 s)	B + MT (7)	B + V (7)	Liver	Immediately after 30% of TBSA burns	10 mg/kg	4-HNE, Nrf2, H0-1
Bekyarova G (2013) [66]	Rat (Male)	220–250	30% of TBSA burns by 90 °C hot water (10 s)	B + MT (8)	B + V (8)	Liver	Immediately after 30% of TBSA burns	10 mg/kg	MDA, TNF-α
Bekyarova G (2012a) [67]	Rat (Male)	220–250	30% of TBSA burns by 98 °C hot water (10 s)	B + MT (7)	B + V (7)	Liver	Immediately after 30% of TBSA burns	10 mg/kg	NF-kB, TNF-α
Bekyarova G (2012b) [68]	Rat (Male)	220–250	30% of TBSA burns by 90 °C hot water (10 s)	B + MT (7)	B + V (7)	Liver	Immediately after 30% of TBSA burns	10 mg/kg	MDA
Bekyarova G (2009a) [69]	Rat (Male)	220–250	20% of TBSA burns by 90 °C hot water (10 s)	B + MT (19)	B + V (19)	Liver	Immediately after 20% of TBSA burns	10 mg/kg	MDA, CRP
Sener G (2002a) [70]	Rat (Both sex)	200–250	30% of TBSA burns by 90 °C hot water (10 s)	B + MT (6)	B + V (6)	Liver, Lung, Intestine	Immediately after 30% of TBSA burns	10 mg/kg	GSH, MDA, PO, MPO
Hristova M (2018) [45]	Rat (Male)	220–250	30% of TBSA burns by 90 °C hot water (10 s)	B + MT (7)	B + V (7)	Gastric mucosa	Immediately after 30% of TBSA burns	10 mg/kg	4-HNE, Nrf2
Hristova M (2016) [71]	Rat (Male)	220–250	30% of TBSA burns by 98 °C hot water (10 s)	B + MT (19)	B + V (19)	Gastric mucosa	Immediately after 30% of TBSA burns	10 mg/kg	MDA, iNOS, HO-1
Hristova M (2015) [72]	Rat (Male)	220–250	30% of TBSA burns by 98 °C hot water (10 s)	B + MT (7)	B + V (7)	Gastric mucosa	Immediately after 30% of TBSA burns	10 mg/kg	MDA
Bekyarova G (2009b) [73]	Rat (Male)	220–250	30% of TBSA burns by 98 °C hot water (10 s)	B + MT (19)	B + V (19)	Gastric mucosa	Immediately after 30% of TBSA burns	10 mg/kg	MDA
Sener G (2002b) [74]	Rat (both sex)	200–250	30% of TBSA burns by 90 °C hot water (10 s)	B + MT Not reported	B + V Not reported	Kidney	Immediately after 30% of TBSA burns	10 mg/kg	MDA, GSH, PO, MPO
Bai XZ (2016) [75]	Rat (Male)	200–250	40% of TBSA burns by 98 °C hot water (12 s)	B + MT (8)	B + V (8)	Kidney	Immediately after 40% of TBSA burns	10 mg/kg	MDA, GSH, SOD, IL-1β, TNF-α, IL-10
Bekyarova G (2010) [76]	Rat (Male)	220–250	30% of TBSA burns by 90 °C hot water (10 s)	B + MT (10)	B + V (10)	Plasma	Immediately after 30% of TBSA burns	10 mg/kg	MDA, CRP
Tunali T (2005) [77]	Rat (Both sex)	200–250	30% of TBSA burns by 90 °C hot water (10 s)	B + MT (6)	B + V (6)	Plasma	Immediately after 30% of TBSA burns	10 mg/kg	MDA, GSH
Al-Ghoul WM (2010) [78]	Rat (Male)	250–300	30% of TBSA burns by (95–97) °C hot water (10 s)	B + MT (3)	B + V (3)	Intestine	Daily for 3 days	1.86 mg/kg	Nitrotyrosine
Han Xiaohua (2006) [79]	Rat (Male)	200–250	30% of TBSA burns by 100 °C hot water (15 s)	B + MT (10)	B + V (10)	Lung	Immediately after 30% of TBSA burns	10 mg/kg	GSH, MDA, GPx, SOD, MPO

Exp = experimental; Con = control; MT = melatonin; TBSA = total body surface area; s = second; B = burn; V = vehicle; 4-HNE = 4-hydroxynonenal; Nrf2 = erythroid 2-related factor 2; TNF-α = Tumor necrosis factor-α; IL-10 = interleukin-10; MDA = malondialdehyde; H0-1 = heme oxygenase-1; NF-kB = nuclear factor kappa-light-chain-enhancer of activated B cells; CRP = C-reactive protein; GSH = glutathione; PO = protein oxidation; MPO = myeloperoxidase; iNOS = inducible nitric oxide synthase; SOD = superoxide dismutase; IL-1β = interleukin-1β; GPx = glutathione peroxidase.

## Supplementary Materials

The following are available online at https://www.mdpi.com/2076-3921/9/12/1196/s1, Supplementary Table S1. Study inclusion criteria. Supplementary Table S2. Explanations for the exclusions of full-text articles. Supplementary Figure S1. Forest plot: leave-one-out sensitivity analysis of the impact of MT on the level of MDA. Supplementary Figure S2. Forest plot: leave-one-out sensitivity analysis of the impact of MT on the level of GSH. Supplementary Figure S3. Forest plot: leave-one-out sensitivity analysis of the impact of MT on the level of 4-HNE. Supplementary Figure S4. Forest plot: leave-one-out sensitivity analysis of the impact of MT on the level of Nrf2. Supplementary Figure S5. Forest plot: leave-one-out sensitivity analysis of the impact of MT on the level of SOD. Supplementary Figure S6. Forest plot: leave-one-out sensitivity analysis of the impact of MT on the level of HO-1. Supplementary Figure S7. Forest plot: leave-one-out sensitivity analysis of the impact of MT on the level of TNF-α. Supplementary Figure S8. Forest plot: leave-one-out sensitivity analysis of the impact of MT on the level of CRP. Supplementary Figure S9. Forest plot: leave-one-out sensitivity analysis of the impact of MT on the level of MPO. Supplementary Figure S10. Forest plot: leave-one-out sensitivity analysis of the impact of MT on the level of IL-10. Supplementary Figure S11. Forest plot: trim and fill analysis.
