# Supplementary material for: Protective Effects of Melatonin against Severe Burn-Induced Distant Organ Injury: A Systematic Review and Meta-Analysis of Experimental Studies
Source: Antioxidants (Basel). 2020 Nov 27;9(12):1196. doi: 10.3390/antiox9121196 (PMC7760393; doi:10.3390/antiox9121196)
Supplement: Supplementary file 1 [file antioxidants-09-01196-s001.pdf]

Supplementary Figures:

Supplementary Fig 1_MDA

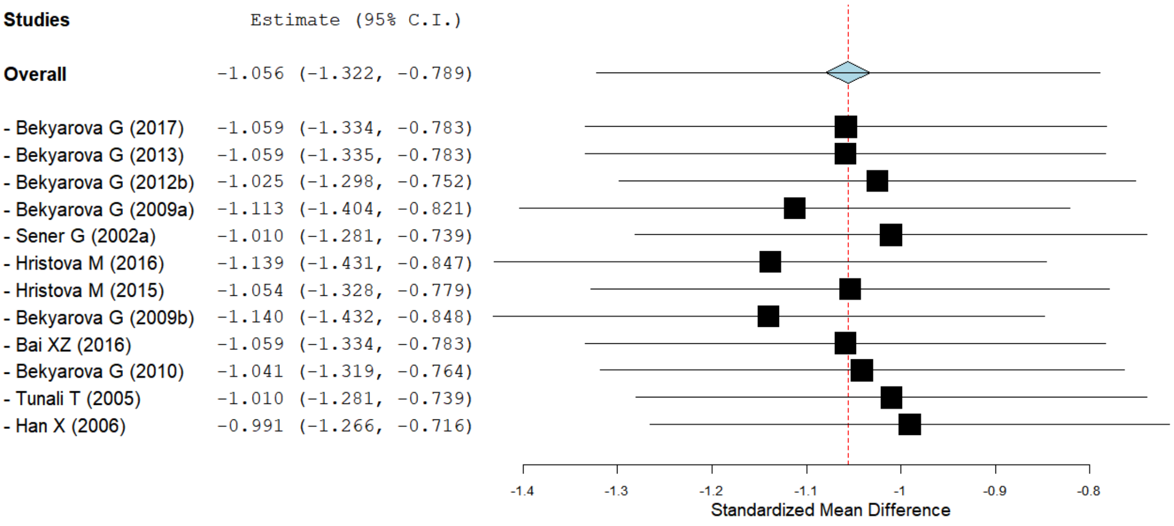

Supplementary Fig 2_GSH

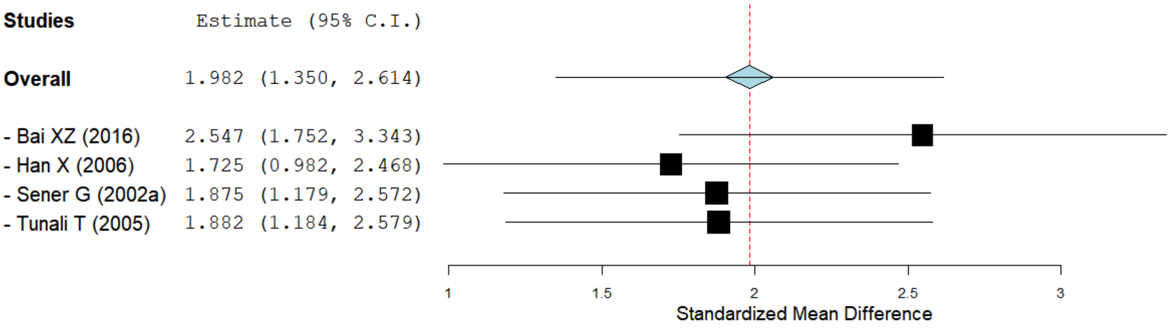

Supplementary Fig 3_4HNE

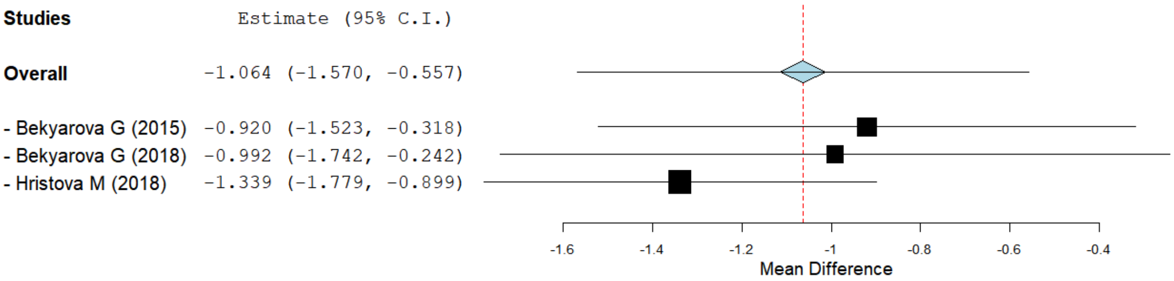

Supplementary Fig 4_Nrf2

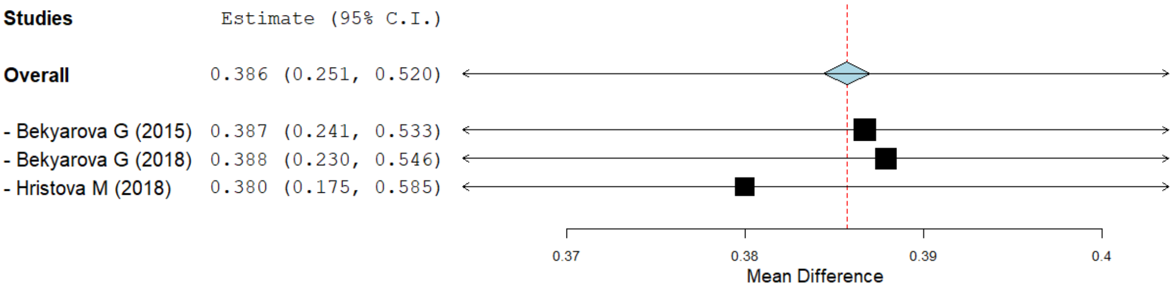

Supplementary Fig 5_SOD

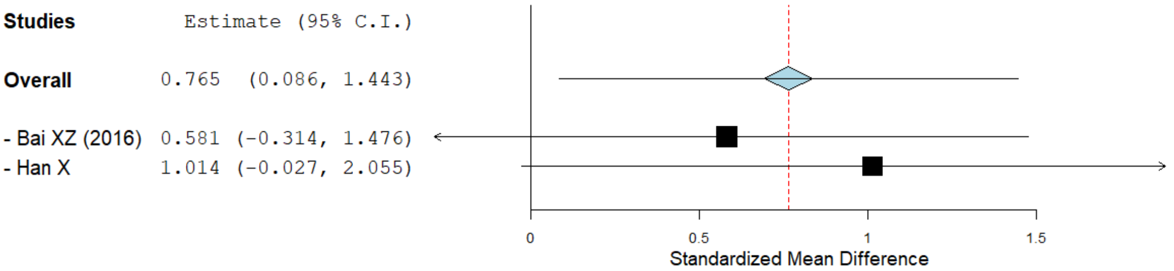

Supplementary Fig 6_HO1

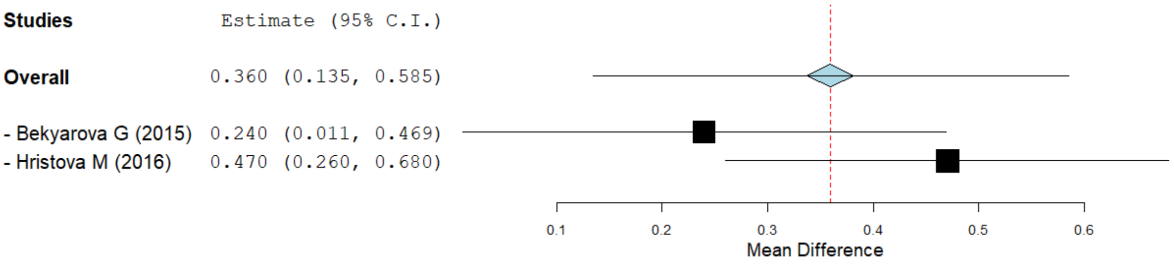

Supplementary Fig 7_TNF-alpha

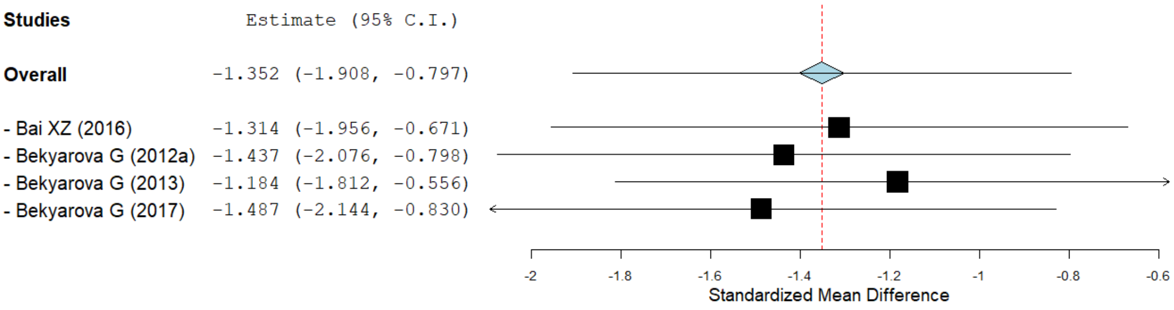

Supplementary Fig 8_CRP

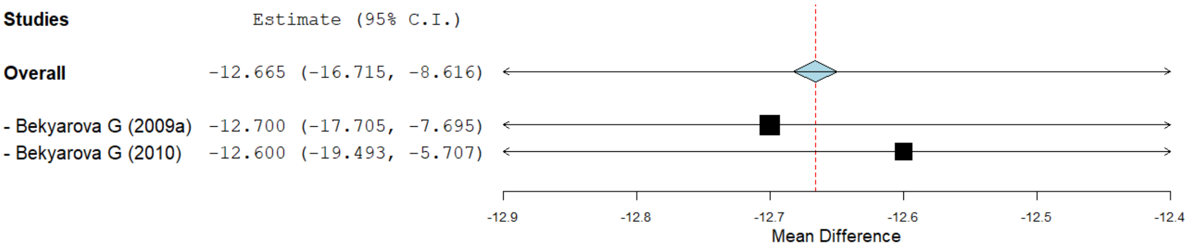

Supplementary Fig 9_MPO

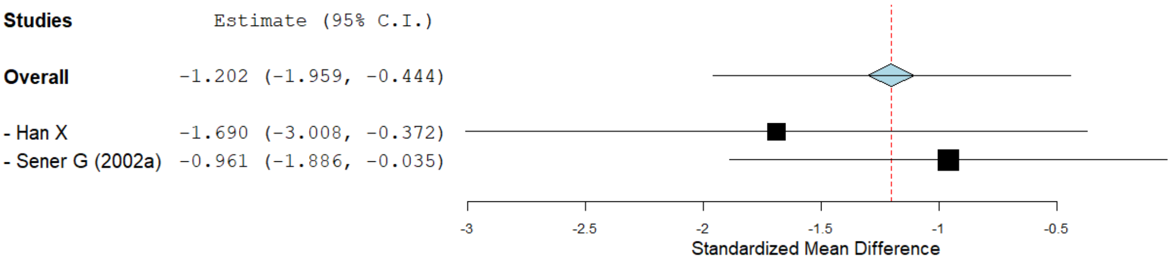

Supplementary Fig 10_IL-10

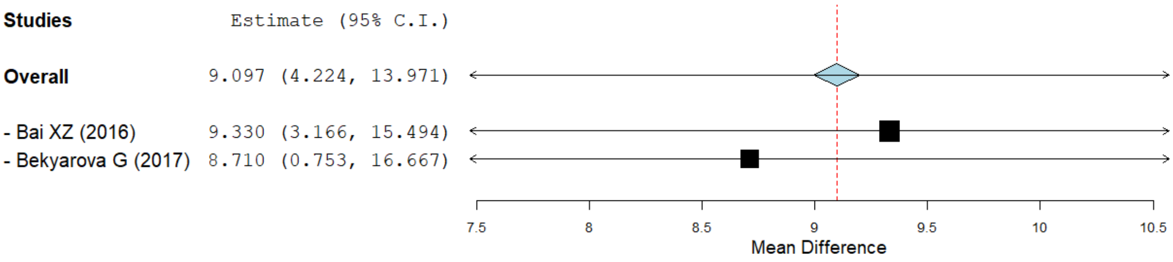

Supplementary Fig 11_trim fill analysis

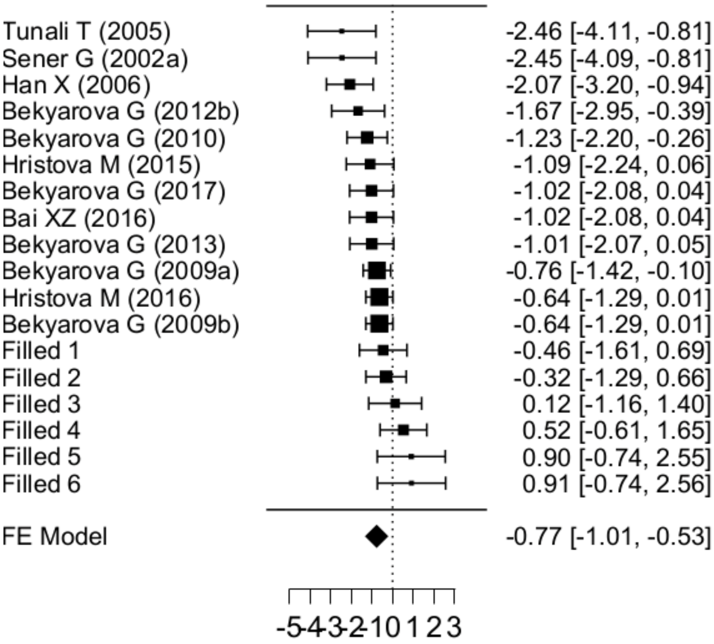

Supplementary Tables:

Supplementary Table S1. Study inclusion criteria.

**Population**	1. Laboratory animals of any sex, age, or strain.<br>2. Burn wound models were established using hot stream/water/surface/wax.<br>3. At least 20-30% total body surface area (TBSA) burns.
**Intervention**	The interventions encompassed any type of melatonin treatment that was compared with a placebo control.
**Comparison**	Any comparator including placebo, saline, ethyl alcohol, or another vehicle.
**Outcomes**	*Primary outcome:*<br>• All possible oxidative stress markers from remote organs after 24 hours of burns. Accumulating evidence suggests that severe burn injury (~30% of the total body surface area or more) induces time-dependent and tissue-specific changes in oxidative stress that persist beyond the acute post-burn period [1]. For example, Szczesny et al. demonstrated that oxidative stress markers such as malondialdehyde (MDA) notably increased at 24 hours in several distant organs like the liver and lung [1].<br>*Secondary outcomes:*<br>• All possible inflammatory markers after 24 hours of burns. The inflammatory response after the initial insult of severe burn persistently increases over a long period of time, leading to multiple organ failure and/or death. For instance, pro-inflammatory cytokines such as tumor necrosis factor-α (TNFα), interleukin-1β (IL-1β), and interleukin-6 (IL-6) levels reached its highest point at 24 hours following severe thermal burn [2,3].

1. Szczesny, B.; Brunyánszki, A.; Ahmad, A.; Oláh, G.; Porter, C.; Toliver-Kinsky, T.; Sidossis, L.; Herndon, D.N.; Szabo, C. Time-Dependent and Organ-Specific Changes in Mitochondrial Function, Mitochondrial DNA Integrity, Oxidative Stress and Mononuclear Cell Infiltration in a Mouse Model of Burn Injury. *PLoS One* **2015**, *10*, e0143730.

2. Finnerty, C.C.; Przkora, R.; Herndon, D.N.; Jeschke, M.G. Cytokine expression profile over time in burned mice. *Cytokine* **2009**, *45*, 20–5.

3. Ipaktchi, K.; Mattar, A.; Niederbichler, A.D.; Hoesel, L.M.; Vollmannshauser, S.; Hemmila, M.R.; Su, G.L.; Remick, D.G.; Wang, S.C.; Arbabi, S. Attenuating burn wound inflammatory signaling reduces systemic inflammation and acute lung injury. *J. Immunol.* **2006**, *177*, 8065–71.

Supplementary Table S2. Explanations for the full-text article exclusions.

SL/NO	Title	Reasons
1	Major thermal injury upregulates the cellular level expression of the melatonin synthesizing enzyme AANAT in gut CD117+ and enterochromaffin cells	Conference Abstract
2	Protective effects of melatonin against caustic esophageal burn injury in rats	Wrong study design
3	Protective effect of melatonin against renal dysfunction, following severe burn in rats	Unavailable
4	Effect of melatonin on wound healing and various biochemical characteristics of granulation-fibrous tissue in rats	Unavailable
5	Melatonin suppresses UV-induced DNA damage represented by cyclobutane pyrimidine dimers formation via enhancing antioxidative enzymes in a human full skin model in vitro	Conference Abstract
6	Murine gut-barrier filamin-a expression: Derangement with major thermal injury and recovery with melatonin treatment	Conference Abstract
7	Effect of melatonin on delayed-type hypersensitivity in severely-burned rats	Wrong outcome
8	Protective effect of melatonin on myocardial injury in severely - Burned rats	Unavailable
9	Melatonin promotes diabetic wound healing in vitro by regulating keratinocyte activity	In-vitro
10	Melatonin inhibits thermal injury-induced hyperpermeability in microvascular endothelial cells	Ex-vivo
11	Saving the zone of stasis in burns with melatonin: an experimental study in rats	Wrong outcome
